# Freeze/Thaw Resistance of Mortar with Recycled Tyre Waste at Varying Particle Sizes

**DOI:** 10.3390/ma16031301

**Published:** 2023-02-03

**Authors:** Riccardo Maddalena

**Affiliations:** School of Engineering, Cardiff University, Cardiff CF24 3AA, UK; maddalenar@cardiff.ac.uk

**Keywords:** mortar, sorptivity, freeze/thaw damage, recycled tyre

## Abstract

There is a growing concern for finding alternative solutions to construction materials in order to minimise their environmental impact as well as enhancing their service life. This study investigated the durability of cementitious mortars prepared by replacing fine aggregate (sand) with recycled tyre shreds and crumbs, aiming at providing an alternative outlet to tyre waste disposal. Tyre shreds obtained at different particle sizes, from fibres of 0.5–5.0 mm to crumbs of 0.1–0.85 mm in diameter, were used as fine aggregate replacement at 20% by volume. The strength of the mortar samples, their thermal conductivity and their water absorption rate were tested at the age of 28 days and after 20 freeze/thaw cycles. The results showed that the mortar containing tyre crumbs at lower particle sizes resulted in negligible shrinkage, improved freeze/thaw resistance, a reduced water absorption by up to 52% and an improved thermal resistivity.

## 1. Introduction

It is estimated that approximately 1.5 billion waste tyres are disposed of annually, and this trend is likely to double in the next 10 years, contributing to a world-wide threat known as ‘black pollution’ [1,2]. Rubber tyres are made of carbon black, elastomers and additives and are reinforced by steel fibres. While efforts have been made in recovering carbon black and fibres from waste tyres, their recovery is otherwise energy-intensive [3,4]. However, tyre waste can be reused as fuel for the production of electricity as well as materials for civil engineering applications, such as road construction, concrete manufacturing and bituminous composites [5,6], providing an alternative outlet to such waste, which is otherwise disposed of in landfill. With the current exponential increase in the demand for construction materials, namely concrete, repurposing end-of-life rubber tyres will not only contribute to diverting millions of waste tyres from landfill disposal but it will also contribute to the production of sustainable construction materials by reducing the amount of natural resources (sand and aggregate) that is extensively used to produce concrete [7,8].

In construction materials, tyre rubber crumbs have been investigated as a replacements for fine aggregate in conventional concrete as well as in high-performance concrete and alkali-activated systems [9,10,11]. Comprehensive review papers on rubberised concrete and mortar illustrated that whilst the addition of rubber tyres particles may reduce the mechanical strength, depending on the aggregate replacement levels, there are beneficial effects such as abrasion resistance, improved durability and carbon-cost savings in the provision of sustainable construction materials [12,13,14,15,16,17].

Ince et al. (2022) investigated the replacement of fine aggregate in concrete with up to 10% by volume of tyre rubber (particle size 0.25–0.50 mm), showing a reduction in compressive strength by 35% [18]. However, when rubber particles were treated with NaOH, their adhesion to the cementitious matrix was improved and led to an increased strength and a mitigated alkali–silicate reaction [19,20,21]. On the contrary, their thermal resistivity performs better with untreated rubber, as it leads to a more porous matrix.

The effect of the adhesion and interfacial bonding between rubber particles and a cementitious matrix was investigated by Huang et al. (2020), where a macro-porosity theory was proposed to assess the strength reduction factor as a function of the rubber particle size and replacement level (up to 80%) [22]. One way to counteract the strength reduction is by combining rubberised concrete with hybrid fibres; this not only improves the overall mechanical strength and impact resistance but it also contributes to the sustainability and circular economy in the building materials sector [23,24]. High levels of aggregate replacement with rubber crumbs have been investigated in numerous studies [25]. Raffoul et al. (2016) highlighted the fact that a rubber content of up to 60% of total aggregate (fine and coarse) is achievable with the use of supplementary cementitious materials (silica fume and pulverised fly ash) to improve the concrete workability and final strength [26]. On the other hand, Richardson et al. (2012) found that low levels of replacement of fine aggregate with rubber crumbs (0.6% by mass) increased the resistance of the concrete to freeze/thaw attack, with minimal scaling compared to the control concrete specimens [27]. Further investigations confirmed that the durability factor is highly influenced by the particle size of tyre crumbs, showing that particles smaller than 0.5 mm offer the maximum frost resistance [1,28,29].

Whilst the addition of rubber tyre particles results in strength loss, it improves the overall durability of mortar, reducing the chloride penetration potential by about 35% with replacement levels at 15% by mass [11]. Other studies have concluded that low percentages of tyre rubber powder up to 20% can decrease the chloride diffusion coefficient compared to control specimens, as well as improving the resistance to carbonation [30,31,32]. The presence of rubber crumbs also has a favourable effect on the thermal resistivity of concrete and mortar, suggesting that such waste can be incorporated into cementitious systems for non-structural applications [17,33,34].

Rubber tyre waste has also been investigated in geopolymers and 3D mortar printing. Rubber particles in alkali-activated cementitious systems showed that a decrease in the compressive strength at replacement levels up to 20% was coupled with an enhanced durability when exposed to sulphate attacks and an improved energy dissipation [10,35]. Such applications have also shown to be promising in carbon emissions costing, as geopolymers are often produced entirely from waste-derived binders [36]. Tyre waste particles and their gradation have been shown to improve the rheological properties of 3D printed mortar, increasing the interlayer bond strength and ductility at the expense of a reduction in the mechanical strength [37,38].

The objective of this study was to assess the effect of the particle size of recycled tyre crumbs on the freeze/thaw resistance of cementitious mortar. An optimal volume fraction of 20% of either rubber shreds or crumbs with a decreasing particle size was chosen. The effect of the varying particle sizes on the physical properties of the mortar samples was evaluated, as well as their strength and shrinkage. The freeze/thaw resistance was investigated by comparing the mechanical strength and physical properties (water transport and thermal conductivity) of the mortar specimens before and after exposure to freeze/thaw cycles.

## 2. Materials and Methods

### 2.1. Materials

Portland limestone cement CEM II/A-L 32.5R was used as the binder. River sand, sieved to a diameter of 2.0 mm, was used as the fine aggregate. Cement and sand were mixed using an orbital mixer at a ratio of 1:3 by mass with unit contents of 480 kg/m^3^ and 1440 kg/m^3^, respectively, and with a water-to-cement (w/c) ratio of 0.5 (240 kg/m^3^), following the mixing procedure outlined in BS EN 196-1:2016 [39].

**Figure 1:**
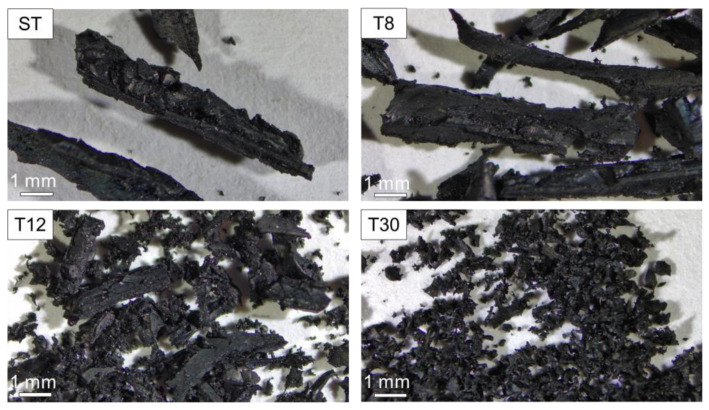
Images of recycled tyre rubber shreds (ST) and rubber crumbs (T8, T12 and T30) at different particle sizes.

Rubber crumbs obtained from the shredding and mechanical grinding of recycled tyres were supplied by SRC Products Ltd. (Stockport, UK). The shreds and crumbs were used as received, with no pre-treatment or washing. Four granulate sizes were selected: coarse rubber shreds (ST), fibres with a typical length of 1.0–5.0 mm and a diameter of 1.0–2.0 mm, fine shreds (T8), acicular granulates with a length of 0.5–2.8 mm and two granulate crumb sizes of 0.4–0.85 mm (T12) and 0.15–0.4 mm (T30), respectively, as shown in Figure 1. The particle size analysis for the crumbs T8, T12 and T30 is provided in Table 1, as supplied by the manufacturer. The bulk density of the rubber shreds (350–500 kg/m^3^) varied with changes in the granulate size.

### 2.2. Methodology

Five series of mortar specimens were prepared by replacing virgin sand with one of the four rubber shreds sizes at a replacement level of 20% by volume (74.3 kg/m^3^) [1] in series ST, T8, T12 and T30, respectively, and their mechanical, physical and thermal properties were compared to a control series (C), without fine aggregate replacement.

After mixing, three samples of each series were cast in standard prismatic moulds (160 mm in length, with a cross-section of 40 × 40 mm) with shrinkage inserts, demoulded after 24 h and then cured in water at 20 °C for 28 days. Shrinkage values were calculated by measuring the length of the prisms with a length comparator device, as detailed in BS EN 12390-16:2019 [40]. The length of the prisms was recorded upon demoulding and after water curing. Samples for thermal conductivity measurements were cast in cylinders (50 mm in diameter and 100 mm in height).

At 28 days, three samples of each series were tested for their flexural strength following the standard BS EN 196-1:2016. After flexural testing, the six broken halves of the three prisms for each series were then subjected to compressive strength, porosity and sorptivity measurements before and after 20 freeze/thaw cycles in saline water, as follows.

Three of the six broken prisms for each series were oven-dried at 60 °C for at least three days or until a stable mass reading was achieved (<0.2%) [41,42]. Open porosity, bulk and matrix density were measured according to the methodology reported in Roels et al. (2004) [43], and the average value was reported. The samples were then conditioned for water absorption rate (sorptivity) measurements on the broken prisms and were then vertically oriented, with the smaller cast face at the bottom, following the methodology adapted from ASTM C1585-13 [44,45,46]. The sorptivity values were calculated as the average of the three samples.

After the water absorption rate measurements, the samples were then tested for their compressive strength following the standard BS EN 196-1:2016, and the average value of the three measurements was recorded.

The thermal conductivity was measured at 20 °C using a Thermtest MP-2 probe (Thermtest Instruments, Hanwell, Canada) on the three cylindrical samples, which were preconditioned at 60 °C for three days according to the standard ASTM D5334-22 [47].

The remaining three of the six broken samples of each series were subjected to 20 freeze/thaw cycles following a methodology adapted from the standard BS EN 12390-9:2016 [48] for concrete. The samples were pre-conditioned at 60 °C for three days and then submerged in a 3% NaCl solution for 12 h. The specimens were then wrapped with polyethylene film and placed in an environmental chamber that simulated a 24 h freeze/thaw cycle by lowering the temperature to –22 °C for 10 h followed by a thawing stage with a constant temperature of +20 °C for 10 h [27]. The samples were subjected to a total of 20 freeze/thaw cycles until significant damage was visible (surface crack formation and scaling). The samples for the thermal conductivity measurements were subjected to the same freeze/thaw protocol, and the thermal conductivity was measured before and after the freeze/thaw cycles.

At the end of the freeze/thaw cycles, the samples were visually inspected for surface damage (crack formation) and scaling. Their mechanical performance (compressive strength) and physical properties (open porosity, density, sorptivity and thermal conductivity) were then measured and compared to those prior to the freeze/thaw attack.

## 3. Results and Discussions

**Figure 2:**
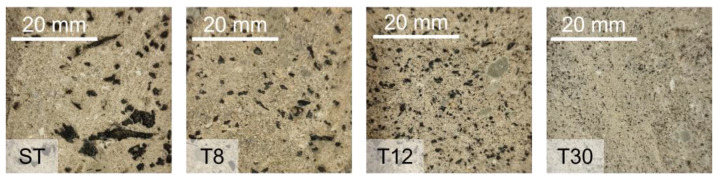
Cross-section of representative mortar samples with rubber shreds (ST) and crumbs at different particle sizes (T8, T12 and T30).

The effects of the rubber tyre shreds and the influence of their particle size is presented in this section. It is important to note that, despite the hydrophobicity of rubber, no bleeding was observed after mixing and casting, nor did any rubber particles rise to the surface, as otherwise suggested by Li et al. (2014) [49]. The rubber particles were uniformly and homogenously distributed across the volume of the samples, as shown in Figure 2.

### 3.1. Effect of Rubber Shreds on Strength and Shrinkage

**Figure 3:**
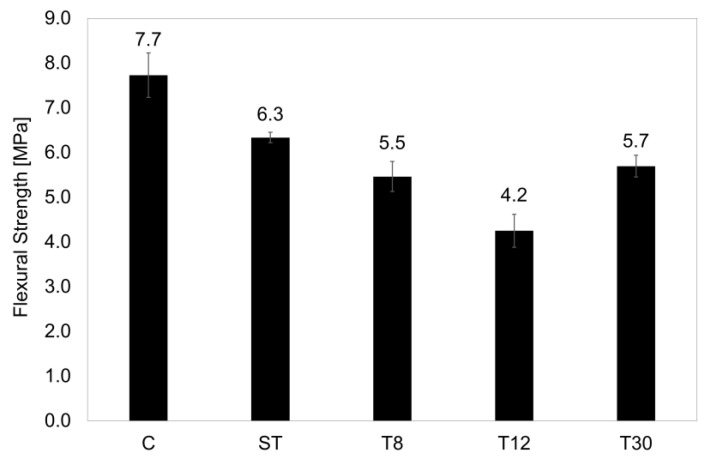
Flexural strength of mortar samples at 28 days of curing. The error bars represent the standard deviation.

The replacement of virgin sand with the rubber shreds reduced the flexural strength of the samples by up to 26% compared to the control sample, as reported in Figure 3. However, the specimens with ST fibres showed a reduction in strength of only 18% from an average of 7.7 MPa for the control series to 6.3 MPa, which was in agreement with the values found in the literature [50,51,52]. This was attributed to the ductile behaviour of the rubber fibres and their aspect ratio (ST fibres) [14]. Decreasing the rubber particle size to <1.0 mm resulted in a further decrease in the flexural strength of 29% and 45%, respectively, for series T8 and T12, which was only to be improved upon by the smaller rubber particles (T30, particle size 0.15–0.40 mm), where the strength loss was 25% lower than the control series. This was due to the particles’ fineness in series T30 and their filler effect [1].

**Figure 4:**
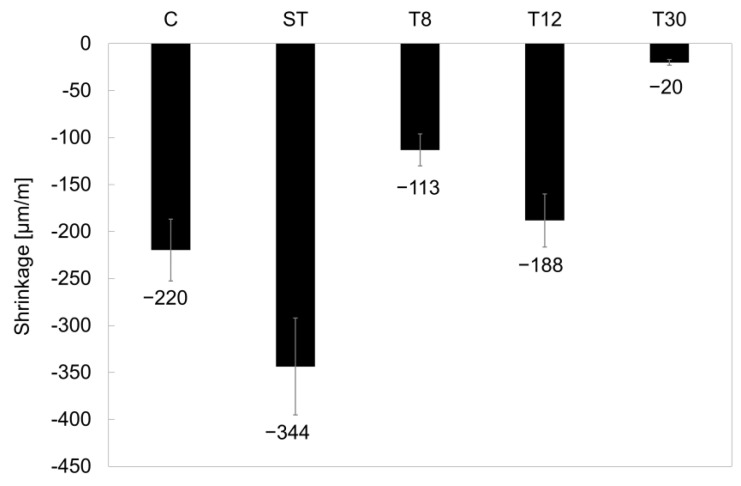
Shrinkage of mortar samples at 28 days of curing. The error bars represent the standard deviation.

The shrinkage measurements for all the series (C, ST, T8, T12 and T30) at 28 days of curing are reported in Figure 4. Except for the samples of series ST, where the shrinkage was approximately 60% larger than the control series, C, the addition of rubber crumbs at lower particle sizes resulted in shrinkage values lower than the control mortar. The ST fibres resulted in the highest shrinkage due to their low stiffness, hydrophobicity and weak bonding with the cementitious matrix, which resulted in reduced internal restraint [17,53].

A change in the tyre particle shape and size, from the fibres in the ST samples to the acicular-shaped particles in series T8, resulted in an improved shrinkage, which was approximately 48% lower than the control series, C, and 67% lower than the larger ST fibres due to increased bonding with the cementitious matrix and high interlocking action [54,55]. On the other hand, when the tyre crumbs were ground into granulate crumbs, the shrinkage reduced with the reduction in the particle size. Series T12 showed an improved shrinkage of −188 µm/m (14% lower than the control series C), whilst series T30 showed a shrinkage of up to 90% lower than the control one (−20 µm/m) due to the lower particle size enhancing the adhesion with the cementitious matrix.

### 3.2. Effect of Rubber Shreds and Crumbs on Freeze/Thaw Resistance

**Figure 5:**
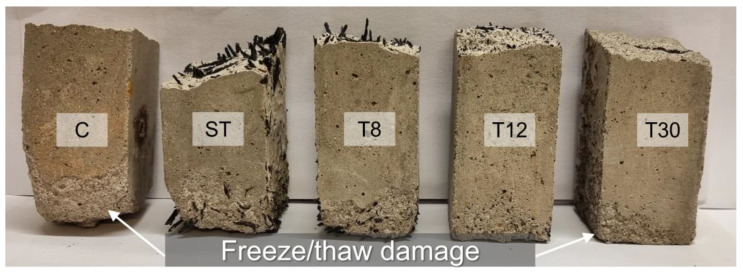
Visual inspection of samples after exposure to freeze/thaw cycles.

The freeze/thaw resistance was assessed by characterising the material’s microstructure (porosity and density), mechanical properties (compressive strength), water absorption rate (sorptivity) and thermal conductivity before and after exposure to freeze/thaw cycles. A visual inspection of the samples (as reported in Figure 5) showed that after 20 cycles of freezing and thawing the addition of rubber shreds and crumbs improved the resistance of the samples to freeze/thaw cycling; the control mortar (C) and the samples in series ST exhibited the most severe surface damage, including scaling and surface cracks, whereas, for the samples with rubber crumbs (series T8, T12 and T30), decreasing the rubber particle size resulted in an increased resistance to scaling (series T30), which was in agreement with previous studies [12,28,31].

#### 3.2.1. Compressive Strength

**Figure 6:**
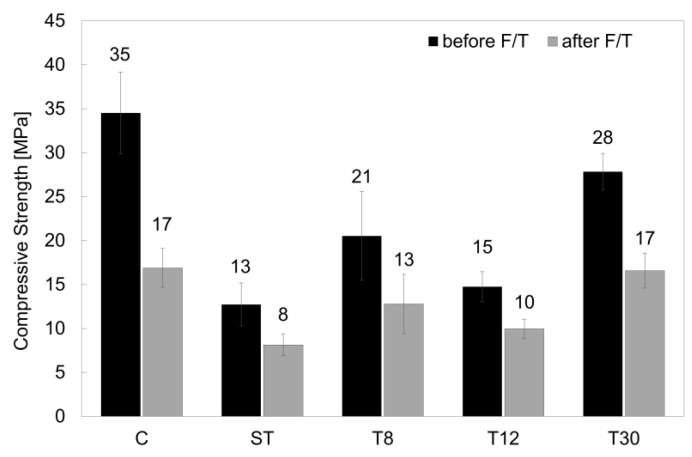
Compressive strengths of series C, ST, T8, T12 and T30 before and after freeze/thaw cycles. The error bars represent the standard deviation.

The rubber shreds and crumbs had a clear effect on the hardened mortar at 28 days, as shown in Figure 6; they reduced the overall strength of the samples, as outlined in previous studies [7,56]. The samples with rubber shreds (ST) showed a 28-day strength of approximately 13 MPa (−60% compared to the control specimens). This was due to the nature of the rubber material as well as its shape, length and hydrophobicity, which prevented sufficient bonding with the matrix [15,49]. However, increasing the particle fineness resulted in a better-performing mortar, with a strength of 28 MPa being obtained when the finer rubber crumbs (T30) were used. Whilst the rubber crumbs affected the strength of the mortar, they had a beneficial effect on the mechanical strength when exposed to frost attacks. After the freeze/thaw cycles, the control specimens showed a decrease in strength of approximately 51% as opposed to an average of 35% when the rubber shreds and crumbs were used. When concrete and mortar are exposed to freeze/thaw cycles, expansion and contraction occurs. The presence of the rubber shreds and crumbs provided a hardened cementitious matrix with air pockets that absorbed the internal damage [8,27]. The lowest particle size used in T30 provided a better mechanical strength after the freeze/thaw cycling damage, which was measured at 17 MPa and was comparable to the control mix, C.

Whilst the compressive strengths for series ST, T8 and T12 were significantly lower than those of the control series C and series T30 (structural strength class), their mechanical performances before and after the freeze/thaw cycles suggested that the rubberised mortar and concrete could be used for non-structural civil engineering applications, such as non-load-bearing elements (walls, hollow blocks, bricks), lightweight concrete, pavements and road applications (asphalt, road barriers) [34,57,58,59,60,61].

#### 3.2.2. Porosity, Density, Sorptivity and Thermal Conductivity

**Figure 7:**
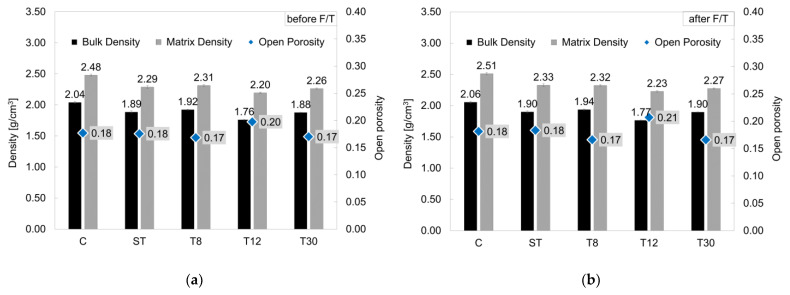
Open porosity, bulk density and matrix density measured (**a**) before and (**b**) after freeze/thaw cycles. The error bars represent the standard deviation.

**Figure 8:**
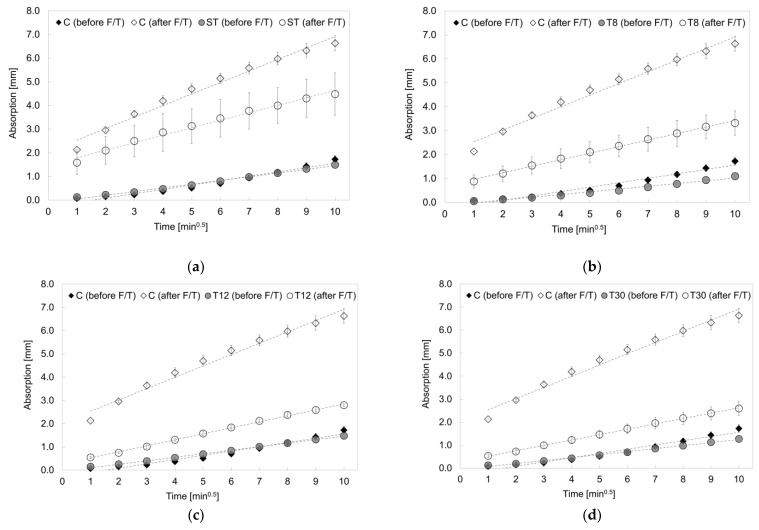
Water absorption rate curves for series (**a**) ST, (**b**) T8, (**c**) T12 and (**d**) T30 before and after freeze/thaw cycles compared to series C. The error bars represent the standard deviation. The dotted lines represent the linear interpolation used to calculate the sorptivity values.

The open porosity and density values measured for all the series were typical of mortar mixes, with the open porosity being in the range of 18–20%, the bulk density being in the range of 1.7–2.0 g/cm^3^ and the matrix density being in the range of 2.2–2.5 g/cm^3^ [62], as shown in Figure 7. The densities (bulk and matrix) were 6–14% lower for the rubber mortars (series ST, T8, T12 and T30) compared to the control mortar, which was due to the lightness of the rubber particles in was agreement with data in the literature [30,63]. However, the freeze/thaw cycles did not have a significant impact on the measured porosity and densities. A slight increase in the density (bulk and matrix) was observed after the freeze/thaw damage (see Figure 7b) due to the formation of cracks and subsequent NaCl salt crystallisation. Whilst this was in contradiction with the visual inspection of the samples shown in Figure 5, where evident damage (scaling and cracks) is visible, the methodology adopted to measure the open porosity and density did not provide the resolution needed to assess the damage to the pore structure. On the other hand, the effect of the freeze/thaw damage was clearly shown in terms of the capillary water absorption rate (sorptivity), which is summarized in Figure 8.

**Figure 9:**
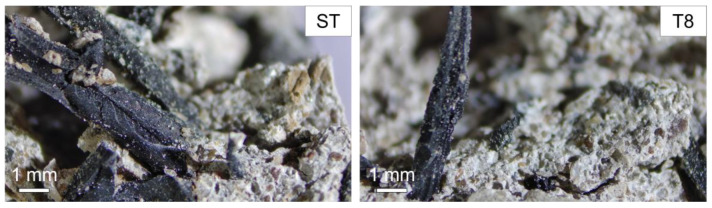
Microscopy images of samples ST and T8 and details of the shreds and fibres, respectively.

The specimens prepared with rubber shreds (series ST) and rubber crumbs (series T8, T12 and T30) showed a total water absorption value at 100 min that was in the range of 13–36% lower than the control specimens (series C), as reported in Figure 8. However, after 20 freeze/thaw cycles, the rubber mortars absorbed less water than the control sample, specifically −32%, −50%, −58% and −61% for series ST, T8, T12 and T30 respectively, showing an increasing resistance to capillary absorption that was proportional to their fineness. This was due to their hydrophobicity and the subsequent weak bonding with the cementitious matrix [22]. The freeze/thaw cycles caused internal damage (i.e., cracks), providing a pathway for water to come into contact with the surfaces of the rubber shreds and crumbs [1,64], as shown in Figure 9.

**Figure 10:**
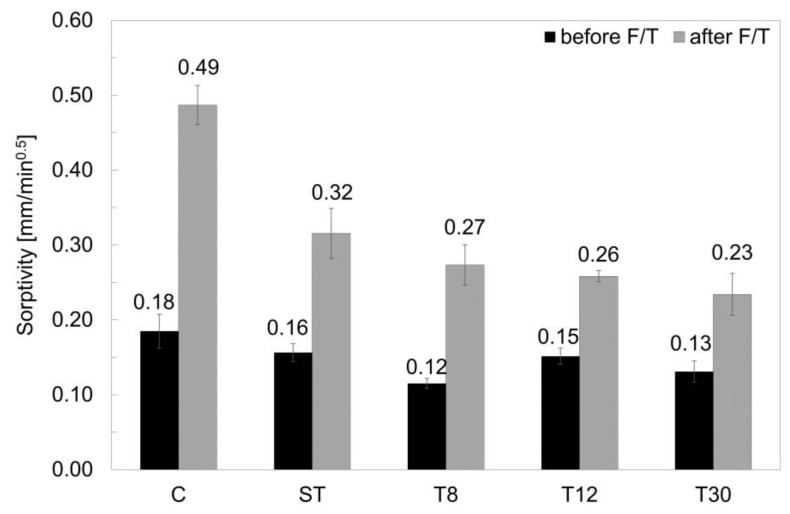
Sorptivity values of series C, ST, T8, T12 and T30 before and after freeze/thaw cycles. The error bars represent the standard deviation.

**Figure 11:**
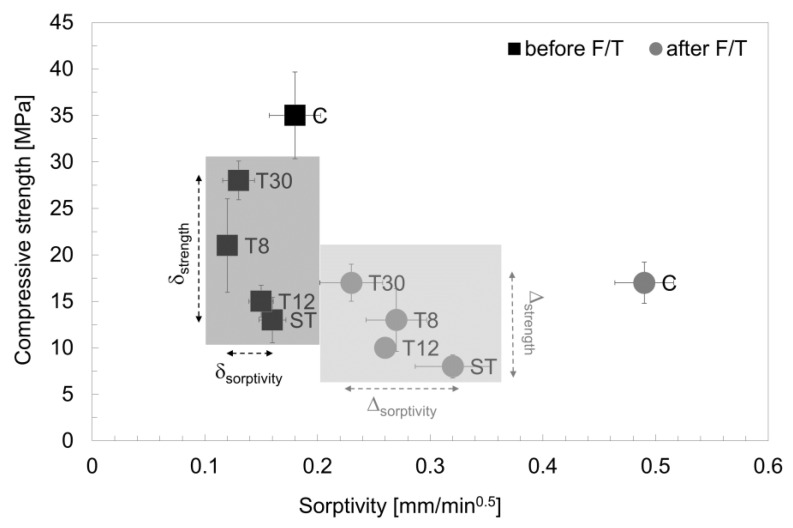
Sorptivity values of series C, ST, T8, T12 and T30 before and after freeze/thaw cycles compared to compressive strength values. The error bars represent the standard deviation.

The calculated sorptivity values before and after the freeze/thaw cycles are shown in Figure 10. It was observed that, before the freeze/thaw damage, the capillary absorption values were within the range of 0.12–0.18 mm/min^0.5^, which is typical of cementitious mortar. However, all the specimens prepared with the recycled tyre waste exhibited an initial sorptivity lower than the control specimens in series C, which was15% for the ST samples, 44% for the T8 samples, 18% for the T12 samples and 29% for the T30 series. Whilst this contradicted the findings of Bisht and Ramana (2017), where a 5% rubber particles replacement led to an increased water absorption [63], the size of the particles used in this study ere up to five-fold lower than those used in Bisht and Ramana (2017), demonstrating the beneficial effect of rubber particle fineness on the capillary absorption of the samples. After the freeze/thaw cycling, the water absorption rate of specimens with rubber shreds and crumbs significantly decreased, with sorptivity values that were 35% (ST), 44% (T8), 47% (T12) and 52% (T30) lower than the control mortars. Furthermore, the decrease in the sorptivity, often used to assess the freeze/thaw resistance, was proportional to the decrease in the particle size, which was in agreement with Zhu et al. (2011) [29]. The same trend was observed when capillary absorption rate values were compared to the mechanical strength, as reported in Figure 11. Whilst before the freeze/thaw damage the larger the particle size, the lower the compressive strength, the strength variation after the freeze/thaw damage was much lower (δ_strength_ > ∆_strength_); the particle size did not affect the sorptivity variation (δ_sorptivity_) before the damage. On the contrary, after the freeze/thaw damage, the capability of the samples to absorb water via capillary action was significantly influenced by the particle size (δ_sorptivity_ < ∆_sorptivity_). The samples prepared with rubber shreds resulted in a higher sorptivity, whereas the variation in strength (∆_strength_) was lower than that before the freeze/thaw damage.

**Figure 12:**
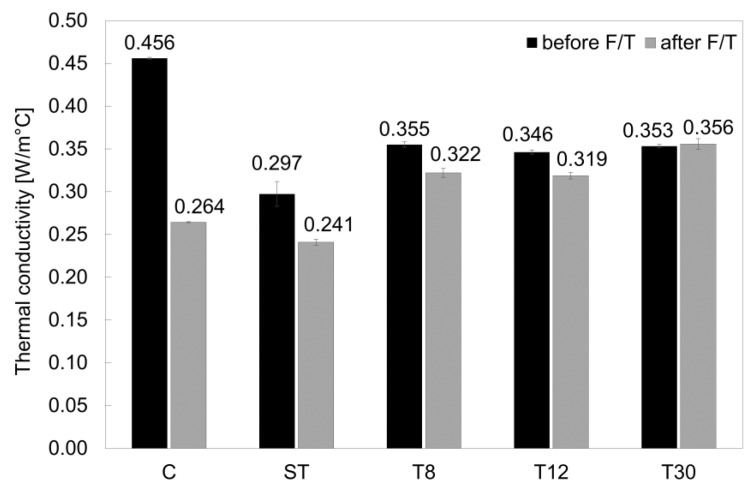
Thermal conductivity values of series C, ST, T8, T12 and T30 before and after freeze/thaw cycles. The error bars represent the standard deviation.

Measurements of the thermal conductivity for all series before and after freeze/thaw damage are reported in Figure 12. The thermal conductivity (λ) of the control mortars (series C) was shown to be typical of cementitious mortar [65,66,67,68]; however, when the rubber shreds and crumbs were used, the λ-value decreased. This was due to the presence of the rubber, a material with a significantly lower density (350–500 kg/m^3^) than mortar constituents (sand and cement). The samples in series ST showed a reduction in thermal conductivity of 35% when compared to the control mortar, whereas the samples in series T8, T12 and T30 reduced the λ-values by approximately 23%, which was in agreement with the literature [69,70]. After the freeze/thaw damage, the formation of internal damage (pores and cracks) further decreased the thermal conductivity in the control samples by 42% due to the presence of air. However, the rubberised samples showed a reduction in thermal conductivity of less than 10% for series T8, T12 and T13 and a reduction of 19% for series ST. The presence of the rubber shreds and crumbs significantly affected the reduction in λ-values, and their particle size aided the freeze/thaw resistance of the system.

#### 3.2.3. Environmental Considerations

The replacement of fine aggregate with rubber tyre waste addresses two key environmental concerns: the extensive use of natural resources in the construction industry and the waste disposal of end-of-life tyres on the other end. Typically, 1 m^3^ of concrete is made up of 350 kg of cement, 700 kg of fine aggregate (sand) and 1200 kg of coarse aggregate (gravel). When scaled-up to large production (thousands of tonnes of concrete per day), partially replacing sand with waste tyre particles, even at relatively low replacement ratios (e.g., 20% by volume) could contribute to saving hundreds of tonnes of sand and subsequent indirect savings in energy and fuel. The embodied carbon (EC) for sand production is estimated to be 0.021 kgCO_2_/kg, which includes the extraction of sandstone or limestone followed by crushing, whereas the EC for tyre waste particles is calculated to be 0.004 kgCO_2_/kg, which accounts for the energy consumption from shredding and grinding [36]. Assuming the production of 1 m^3^ of mortar with sand replacement levels of up to 50–80% with recycled tyre waste [22], the EC of mortar with fine aggregate would be 20–30% lower than conventional mortar.

Tyre waste disposal also represents a growing environmental concern, as landfill disposal is costly and not environmentally sustainable; in the UK, only 10% of tyre waste is recycled, and 63% is disposed of in landfill [2]. Tushar et al. (2022) calculated that recycling the annual waste disposal of 460,000 tonnes for the production of rubber crumbs could save up to $16.1 million in natural resources [6]. Repurposing waste tyres into cementitious materials would divert hundreds of thousands of tonnes of tyres from landfill disposal towards being reused in the construction industry [1].

The results discussed in this work link two large and growing industries (concrete manufacturing and tyre production) by providing waste materials with a way to replace sand on the one hand and alternative solutions to tyre waste disposal on the other.

## 4. Conclusions

In this study, the freeze/thaw resistance of rubberised mortars was evaluated by means of mechanical performance tests, water transport measurements and thermal conductivity measurements. The fine aggregate (sand) in the mortar samples was replaced with 20% (by volume) of waste tyre shreds and crumbs. The effects of their particle size (from fibres 0.5–5.0 mm long to crumbs of 0.15–0.85 mm in diameter) was shown to influence the overall mechanical performance of the samples. The flexural strength and compressive strength values were reduced by approximately 18–45% and 20–62%, respectively, when compared to the control specimens. However, the smaller particle size of the tyre crumbs improved the drying shrinkage by approximately 90%, i.e., −20 μm/m for series T30 compared to −220 μm/m for the control series.

The addition of waste tyre particles significantly improved the mortar’ freeze/thaw resistances. After the freeze/thaw damage, decreasing the tyre particle size resulted in an improved water absorption rate by up to 50% when compared to the plain mortar. A similar trend was observed in the measured thermal conductivity before and after 20 cycles of freezing and thawing, where the specimens with a smaller particle size showed the lowest variation in λ-values.

The results presented in this work suggest that the particle size of tyre crumbs plays an important role in using waste tyres for mortar and concrete production for non-structural applications; the optimisation of the replacement levels and grading leads to improved durability frost resistance, and enhanced thermal resistivity.

Further work will be conducted at the concrete scale, including microstructural characterisations and a detailed life-cycle assessment to evaluate the environmental benefits in targeted engineering applications.

## Figures and Tables

**Table 1:** Particle size analysis for recycled tyre crumbs T8, T12 and T30.

Test Sieve	T8	T12	T30	Test Sieve	T8	T12	T30
mm	% Retained mass			mm	% Retained mass		
3.35	–	–		0.71	–	10–25	0–0.1
2.80	0–5	–		0.50	10–30	–	–
2.36	0–15	–		0.425	–	10–25	0–1
1.70	10–30	0–0.5		0.355	–	–	–
1.18	–	10–40		0.25	–	0–5	40–60
1.00	40–60	–		0.15	–	–	25–35
0.85	–	30–45		Pan	0.5	0–5	0–15

## Data Availability

The data associated with this work are available under the Cardiff University Dataset repository, and can be accessed at http://doi.org/10.17035/d.2022.0235158295.
